# MicroRNA-4732-3p Is Dysregulated in Breast Cancer Patients with Cardiotoxicity, and Its Therapeutic Delivery Protects the Heart from Doxorubicin-Induced Oxidative Stress in Rats

**DOI:** 10.3390/antiox11101955

**Published:** 2022-09-30

**Authors:** Rafael Sánchez-Sánchez, Ignacio Reinal, Esteban Peiró-Molina, Marc Buigues, Sandra Tejedor, Amparo Hernándiz, Marta Selva, David Hervás, Antonio J. Cañada, Akaitz Dorronsoro, Ana Santaballa, Carmen Salvador, Florian Caiment, Jos Kleinjans, Luis Martínez-Dolz, Isabel Moscoso, Ricardo Lage, José R. González-Juanatey, Joaquín Panadero, Ernesto Aparicio-Puerta, Antonio Bernad, Pilar Sepúlveda

**Affiliations:** 1 Regenerative Medicine and Heart Transplantation Unit, Instituto de Investigación Sanitaria La Fe, 46026 Valencia, Spain; 2 Data Science Unit, Instituto de Investigación Sanitaria La Fe, 46026 Valencia, Spain; 3 Clinical and Translational Research in Cancer, Instituto de Investigación Sanitaria La Fe, 46026 Valencia, Spain; 4 Department of Toxicogenomics, School of Oncology and Developmental Biology (GROW), Maastricht University, 6211 LK Maastricht, The Netherlands; 5 Clinical and Translational Research Group in Cardiology, Instituto de Investigación Sanitaria La Fe, 46026 Valencia, Spain; 6 Centro de Investigación Biomédica en Red Enfermedades Cardiovasculares (CIBERCV), Carlos III Institute of Health, 28029 Madrid, Spain; 7 Cardiology Group, Center for Research in Molecular Medicine and Chronic Diseases (CIMUS), University of Santiago de Compostela, 15782 Santiago de Compostela, Spain; 8 Department of Cardiology and Coronary Unit, Institute of Biomedical Research (IDIS-SERGAS), University Clinical Hospital of Santiago de Compostela, 15782 Santiago de Compostela, Spain; 9 IGENOMIX, 46980 Valencia, Spain; 10 Chair for Clinical Bioinformatics, Saarland University, 66123 Saarbrücken, Germany; 11 Department of Immunology & Oncology, National Center for Biotechnology (CNB-CSIC), Campus de Cantoblanco de la Universidad Autónoma de Madrid, 28049 Madrid, Spain

**Keywords:** cardiotoxicity, doxorubicin, miR-4732-3p, oxidative stress, fibrosis, angiogenesis, cardiac function, TGFβ pathway, Hippo signaling pathway

## Abstract

Anthracycline-induced cardiotoxicity is the most severe collateral effect of chemotherapy originated by an excess of oxidative stress in cardiomyocytes that leads to cardiac dysfunction. We assessed clinical data from patients with breast cancer receiving anthracyclines and searched for discriminating microRNAs between patients that developed cardiotoxicity (cases) and those that did not (controls), using RNA sequencing and regression analysis. Serum levels of 25 microRNAs were differentially expressed in cases versus controls within the first year after anthracycline treatment, as assessed by three different regression models (elastic net, Robinson and Smyth exact negative binomial test and random forest). MiR-4732-3p was the only microRNA identified in all regression models and was downregulated in patients that experienced cardiotoxicity. MiR-4732-3p was also present in neonatal rat cardiomyocytes and cardiac fibroblasts and was modulated by anthracycline treatment. A miR-4732-3p mimic was cardioprotective in cardiac and fibroblast cultures, following doxorubicin challenge, in terms of cell viability and ROS levels. Notably, administration of the miR-4732-3p mimic in doxorubicin-treated rats preserved cardiac function, normalized weight loss, induced angiogenesis, and decreased apoptosis, interstitial fibrosis and cardiac myofibroblasts. At the molecular level, miR-4732-3p regulated genes of TGFβ and Hippo signaling pathways. Overall, the results indicate that miR-4732-3p is a novel biomarker of cardiotoxicity that has therapeutic potential against anthracycline-induced heart damage.

## 1. Introduction

Anthracyclines are effective and extensively used antineoplastic drugs for the treatment of a broad range of malignancies, including breast cancer [1]. Unfortunately, their use is associated with several adverse effects, amongst which cardiotoxicity is considered one of the most severe [2,3]. In people treated with anthracyclines, cardiotoxicity symptoms can appear within days, during the first year after administration of the last dose or even after 6–10 years [4]. Accordingly, the early detection of anthracycline-induced cardiotoxicity could prevent irreversible heart injury by helping in the assessment of the risk–benefit balance of chemotherapy [5,6]. The most common approaches for monitoring cardiac damage are the evaluation of left ventricular ejection fraction (LVEF) and global longitudinal strain, which can precede deteriorations in cardiac function. Other endpoints include blood biomarkers such as troponins (I and T), B-type natriuretic peptide (BNP) and N-terminal BNP (NT-proBNP) [7]. More recently, microRNAs (miRNAs) have entered the clinical arena as potential biomarkers of anthracycline-induced cardiotoxicity [8,9,10,11,12].

Circulating miRNAs are non-coding RNAs of 20–25 nucleotides that play influential roles in the post-transcriptional ‘fine-tuning’ of gene expression. The binding of mature miRNAs to their mRNA targets negatively influences the expression of specific proteins, either by degradation of the bound mRNA target or by translational inhibition. They are present in many human fluids, such as serum and plasma, show high stability and are involved in diverse pathological processes including cardiovascular disease [13,14] and cancer [15]. Consequently, miRNAs and other non-coding RNAs are being investigated as biomarkers for cardiotoxicity [10] and as therapeutic targets [16,17,18,19]. A recent meta-analysis of 209 studies reporting miRNA biomarkers of cardiotoxicity in patients with breast cancer found that let-7f, miR-1, miR-20a, miR-126 and miR-210 were the more consistently dysregulated in patients, and the authors proposed that miR-1 might serve as a robust biomarker of heart failure [20].

In the present study, we prospectively followed a cohort of patients with breast cancer receiving anthracyclines for five years in a multicenter study and evaluated cardiac function at baseline and during the first year following completion of chemotherapy [4]. Using RNA sequencing (miRNAseq) analysis of serum samples after chemotherapy, we obtained a panel of 25 miRNAs that were differentially expressed in patients who experienced a decline in cardiac function. From this panel, we identified miR-4732-3p as a biomarker downregulated in serum and plasma in patients that developed cardiotoxicity after chemotherapy.

We have previously described that miR-4732-3p is modulated by hypoxia [21]. Notably, gain of function experiments using an miRNA-4732-3p mimic prolonged spontaneous beating and reduced reactive oxygen species (ROS) levels in oxygen- and glucose-deprived neonatal rat cardiomyocytes (NRCM). Additionally, miRNA-4732-3p induced angiogenic responses and preserved cardiac function when injected intramyocardially in an experimental model of myocardial infarction.

We assessed the cardioprotective effect of miR-4732-3p against doxorubicin damage, finding that it induced anti-apoptotic responses and inhibited oxidative damage both in NRCM and cardiac fibroblasts (cFib) treated with doxorubicin. It also prevented weight loss, reduced fibrosis and preserved cardiac function in a rat model of doxorubicin-induced cardiotoxicity. Overall, our findings suggest that miR-4732-3p is a circulating novel biomarker of early stages of cardiotoxicity and we provide evidence that miR-4732-3p overexpression protects against heart failure.

## 2. Materials and Methods

### 2.1. Patients

The prospective observational case-control study was conducted at Hospital Universitari i Politècnic La Fe (HUiPLaFe). Serial patients with breast cancer that had undergone breast surgery from 2013–2018 in that hospital were enrolled before the initiation of chemotherapy (adjuvant therapy). All patients were >18 years of age at the time of inclusion and provided written informed consent. The main cohort comprised 10 patients with breast cancer that experienced a decline in cardiac function during the first year after completion of anthracycline-based chemotherapy (cases) and 10 matched patients that did not (controls). Serum samples were obtained after the completion of treatment. To consolidate the findings from the main cohort, we analyzed a second cohort of patients with breast cancer and treated at another hospital (Hospital Clínico de Santiago de Compostela, HCSC) with the same anthracycline-based adjuvant chemotherapy. This validation cohort was a plasma sample collection from 32 patients with breast cancer (7 cases and 25 controls) obtained retrospectively.

### 2.2. Clinical Data Collection

Clinical data were gathered from the medical records of patients before and at different periods after completion of treatment. Data included demographics, medical history, cardiovascular risk factors, use of concomitant medication and/or antineoplastic regimens, symptoms and signs of cardiovascular disease, results of physical examination, echocardiography and routine laboratory results.

### 2.3. Main Study Parameters/Endpoints

The primary endpoint was the development of cardiotoxicity, defined by the most recent clinical guidelines as one of the following findings on transthoracic echocardiography: a 5% reduction in LVEF to a final LVEF <55% with symptoms of heart failure, or a reduction of 10% to a final LVEF <55% in asymptomatic patients [22,23].

### 2.4. Echocardiography in Patients

Echocardiography was performed before and after the chemotherapeutic regimen and at 12 months after the end of the treatment. Conventional echocardiography consisted of two-dimensional measurements to assess LV structure and global function. All measurements were taken in accordance with the current guidelines of the American Society of Echocardiography using the iE33 scanner (Philips Medical Systems, Andover, MA) with transthoracic S5-1 and X5-1 broadband transducers (frequency = 1–5 MHz). The parasternal long-axis view was used to measure septal and posterior wall thickness and LV systolic and diastolic diameters (LVESD and LVEDD, respectively), and volumes were calculated using Teicholz’s equation (LVV = (7/(2.4 + LVD) × LVD^3^). Cross-sectional images were recorded from the apex, and end-diastolic and end-systolic areas and LV lengths were measured from the apical four-chamber (A4C) and two-chamber (A2C) views (using the modified biplane Simpson’s method) for the calculation of EF. All tracings were made at a centralized reading center by a single observer who was blinded to all other clinical and biomarker data.

### 2.5. Sample Collection

Serum samples from the main cohort were taken just before the first dose of chemotherapy and at different intervals after completion of treatment. Blood was drawn into non-heparinized tubes, and serum was separated after clotting for 2 h. Samples were stored at the HUiPLaFe Biobank and were processed following standard operating procedures with the appropriate approval of the ethics and scientific committees. Blood samples from the validation cohort were collected in heparinized tubes, and plasma was harvested and stored at the HCSC Biobank.

### 2.6. Animal Ethics Statements

Animal procedures were approved by the institutional ethical and animal care committees according to guidelines from Directive 2010/63/EU of the European Parliament on the protection of animals used for scientific purposes, enforced in Spanish law under Real Decreto 1201/2005 (GVA authorized procedures 2019/VSC/PEA/0249 for Wistar colony maintenance and 2019/VSC/PEA/0260 for a rat model of doxorubicin-induced cardiotoxicity).

### 2.7. Primary Cell Cultures and Cell Lines

NRCMs were isolated as described by Armiñán et al. [24], with some modifications. Briefly, rats aged 0 to 2 days were euthanized by decapitation, and the hearts were extracted, minced and incubated overnight in Hanks’ Balanced Salt Solution (HBSS, without Ca and Mg) with 0.05% trypsin (Sigma-Aldrich, Madrid, Spain) at 4 °C and gentle agitation. Subsequently, the heart fragments were digested for 40 min at 37 °C in an enzyme mixture (0.04% collagenase, 0.02 mg/mL DNase) in Leibovitz L15 medium (all from Gibco-Invitrogen, Grand Island, NY). The first cell suspension was discarded, and the fragments were digested for 20 min in the same enzyme mixture. This step was repeated until there were no tissue fragments. Each cell suspension was collected and centrifuged at 90× *g* for 5 min at room temperature (RT), and the cells were resuspended in plating medium composed of Dulbecco’s Modified Eagle Medium (DMEM) containing 15% fetal bovine serum (FBS), 20% M-199 Medium and 1% penicillin/streptomycin (all from Gibco-Invitrogen). The cells were then incubated for 1.5 h under standard culture conditions (37 °C in 5% CO_2_) to allow fibroblasts to attach to the culture plates. Then, the medium containing unattached cells was centrifuged and cardiomyocytes were pelleted and seeded in 0.1% gelatin-coated plates using plating medium. The following day the cardiomyocytes were washed three times with PBS and maintained in DMEM supplemented with 20% M-199 Medium, 4% horse serum (HS, Gibco-Invitrogen) and 1% penicillin/streptomycin.

For cardiac fibroblast (cFib) isolation, hearts from rats aged 0 to 2 days were extracted, minced and incubated in DMEM F-12 medium (Gibco-Invitrogen) with 0.075% trypsin for 10 min at 37 °C. The supernatant was discarded, and heart fragments were incubated with an enzyme mixture of 0.2% collagenase II and 0.01 mg/mL DNase I in DMEM F-12 medium for 30 min at 37 °C. Then the cell suspension was collected and centrifuged at 300× *g* for 5 min. The cFib medium was then resuspended in DMEM F-12 medium supplemented with 10% FBS and 1% penicillin/streptomycin. The next day the cFib medium was changed, and cells were maintained with DMEM F-12 medium supplemented with 10% FBS and 1% penicillin/streptomycin.

Human cardiac fibroblasts (HCF) were purchased from PromoCell^®^ (Heidelberg, Germany) and cultured in Fibroblast Growth Medium 3 (PromoCell^®^). Human induced pluripotent stem cell cardiomyocytes (hiPSC-CMs) (iCell Cardiomyocytes, Cellular Dynamics, Wisconsin, USA) were thawed and cultured according to manufacturer’s instructions. Human embryonic kidney cells 293 (HEK 293) were cultured in DMEM supplemented with 10% FBS and 1% penicillin/streptomycin.

### 2.8. Cell Transfection, Cell Transduction and Doxorubicin Treatment

To test whether the selected miRNAs showed a cardioprotective effect against doxorubicin, we established a transfection protocol using corresponding synthetic miRNA mimics (MISSION miRNA Mimic, Merck KGaA, Darmstadt, Germany) and doxorubicin treatment. NRCM or cFib, HCF or hiPSC-CMs were seeded and incubated for 2–3 days. The mimics of hsa-miR-4732-3p or cel-miR-243-3p from *Caenorhabditis elegans* were then transfected individually at a final concentration of 40 nM using Lipofectamine 3000 (ThermoFisher Scientific, Waltham, MA, USA). Equal volumes of mimic diluted in OptiMem (ThermoFisher Scientific) and Lipofectamine 3000 1:25 in OptiMem were mixed and allowed to form Lipofectamine-mimic complexes for 15 min at RT. The Lipofectamine-mimic complexes were then added to the cells to give a final mimic concentration of 40 nM, and cells were incubated for 24 h. To induce cardiac damage, the cells were treated with 1 µM doxorubicin for 48 h to imitate an acute dose of doxorubicin. To induce stable expression of miR-4732-3p and to validate target genes, HEK 293 cells were transduced with LentimiRa-GFP-hsa-miR-4732-3p Vector (Abm, Vancouver, BC, Canada), and cells expressing the vector were selected using puromycin at 1 μg/mL.

### 2.9. RNA Sequencing

For RNAseq analysis of blood from patients (main cohort), total RNA was first isolated from serum using the miRNeasy Mini Kit (Qiagen, Westburg BV, Leusden, The Netherlands). Small RNAs were size-selected and ligated using the TruSeq Small RNA Library Preparation Kit (Illumina, San Diego, CA, USA). Samples were sequenced on the HiSeq 2000 platform (Illumina) as single-end 50-bp reads, with an average of 4.6 million reads. Raw reads were trimmed to remove the adapter sequence and were mapped using Patman [25] against the known human miRNA sequence from miRbase (release 21, http://www.mirbase.org (accessed on 13 January 2018)).

### 2.10. qPCR Detection of miRNAs in Blood Samples and Cell Cultures

Reverse transcription was performed with the miRCURY LNA Universal RT miRNA PCR Kit (Qiagen, Madrid, Spain). The miRNA was quantified using a Viia TM 7 Real System and the results were analyzed with QuantStudio Real-Time PCR Software (Applied Biosystems, Foster City, CA, USA).

### 2.11. RT-qPCR

RNA was isolated from cells using the RNeasy Plus Mini Kit (Qiagen, Westburg BV, Leusden, The Netherlands). cDNA was obtained by reverse transcription employing the PrimeScript RT Reagent Kit (Takara, Kusatsu, Japan). RT-qPCR was performed with the respective rat-specific sense and antisense primers and TB Green^®^ Premix Ex Taq^TM^ (Tli RNase H Plus, Takara, Kusatsu, Japan). The primers used were:

*Gapdh* (NM_017008.4) CAACACTGAGCATCTCCCTCAC (F) TATTCGAGAGAAGGGAGGGCT (R)

*Nfe2l2* (NM_031789.2) GCCCTGGATATTCCCAGCC (F) TCTCAGCCTGCTGCTTGTTT (R)

*Nrf1* (NM_001100708.1) TACAAGGCGGGGGACAGATA (F) TGCATGAACTCCATCTGGGC (R)

### 2.12. Western Blotting

Cells were lysed in RIPA buffer (1% NP40, 0,5% deoxycholate, 0,1% sodium dodecyl sulfate in Tris-buffered saline (TBS)) (Sigma-Aldrich) supplemented with protease (Complete, Sigma-Aldrich) and phosphatase (PhosSTOP, Sigma-Aldrich) inhibitors. Equal amounts of samples were mixed with non-reducing Laemmli sample buffer (BioRad, Hercules, CA, USA) and denatured at 96 °C for 5 min. Proteins were separated on 10% SDS-polyacrylamide gels and transferred to polyvinylidene difluoride membranes (Immobilon-P; Millipore, Bedford, MA, USA). Membranes were blocked with 5% non-fat dry milk powder in TBS. Primary antibodies used for western blotting were: anti-Smad2 (1/500, Abcam, Cambridge, UK, ab228765), anti-YAP (1/1000, Cell Signaling Technology, Danvers, MA, USA, D8H1X), anti-phospho-YAP (1/1000, Cell Signaling Technology, D9W2I) and anti-GAPDH (1/1000, Cell Signaling Technology, D16H11). Secondary antibody was anti-IgG rabbit (1/5000, Dako, Santa Clara, CA, USA, P0448). Detection was carried out using peroxidase-conjugated antibodies and SuperSignal^TM^ West Femto (Thermo Fisher Scientific). Reactions were visualized using an Amershan Imager 600 (GE Healthcare, Chicago, IL, USA) and quantified with ImageJ software (NIH, Bethesda, MD, USA).

### 2.13. Cell Viability CCK8 Assay

Cell viability was measured in cell cultures using the Cell Counting Kit-8 (CCK8; Merck KGaA, Darmstadt, Germany). Cells were incubated with 10% CCK8 reagent in the corresponding medium for 3 h at 37 °C. Absorbance was then measured at 450 nm using a microplate reader (Halo LED 96; Dynamica Scientific, Newport Pagnell, UK). Data were analyzed using Prism 8.0 software (GraphPad Software Inc., La Jolla, CA, USA).

### 2.14. Annexin-V Staining

Doxorubicin-treated cells were detached with trypsin/EDTA, centrifuged at 1800 rpm for 5 min, resuspended in binding buffer with annexin-V-FITC and DAPI (R&D Systems, Minneapolis, MN, USA) and incubated for 15 min at RT. The percentage of annexin-positive cells was measured by flow cytometry in a FACS Canto II cytometer (BD Bioscience, San Jose, CA, USA).

### 2.15. Lactate Dehydrogenase Assay

Supernatants of NRCM cultures were tested for lactate dehydrogenase using the Cytotoxicity Detection KitPLUS (LDH) (Roche, Indianapolis, IN, USA).

### 2.16. Oxidative Stress Assessment In Vitro

Cells were washed with PBS and stained either with 5 μM DCFH-DA (2′,7′-dichlorofluorescin diacetate; Sigma-Aldrich) for 30 min, for whole cell reactive oxygen detection, or with 5 μM MitoSOX^TM^ Red (ThermoFisher Scientific) for 30 min, for mitochondrial superoxide detection. After staining, cells were washed three times with PBS, detached with trypsin/EDTA and analyzed by flow cytometry in a FACS Canto II cytometer (BD Bioscience). Data were analyzed using FlowJo X (TreeStar Inc., Ashland, OR, USA).

### 2.17. Experimental Model of Doxorubicin Induced Cardiotoxicity and Histological Analysis

#### 2.17.1. Animals

Wistar rats weighing 200–250 g (Charles River Laboratories Inc., Wilmington, MA, USA) were used in an in vivo model of doxorubicin-induced cardiotoxicity. The initial number of animals included in the study was 14. Mortality due to doxorubicin treatment was ~20%.

#### 2.17.2. Doxorubicin Treatment and In Vivo Administration of miR-4732-3p

In total, 14 male rats were randomly divided into two experimental groups. The first group received a total of 7 intraperitoneal injections (once weekly) of doxorubicin hydrochloride (D2975000, Merck, KGaA, Darmstadt, Germany) at 2 mg/kg, for 7 weeks, giving an accumulative dose of 14 mg/kg. The second group received the same schedule of doxorubicin and also received miR-4732-3p encapsulated in a liposomal emulsion intravenously in the second and fourth week. To prepare the emulsion, 16 µL of miR-4732-3p was mixed with 8.5 µL of the Maxsuppressor in vivo RNA-LANCEr-II lipidic Reagent (Cosmo Bio Co., Ltd., Tokyo, Japan). The lipid formulation was injected in the tail vein at weeks 2 and 4 after the initial boost of doxorubicin. The final dose of 4732-mimic was 70 μg/rat.

#### 2.17.3. Echocardiography in Rats

Transthoracic echocardiography was performed under inhalational anesthesia (Sevorane^TM^, AbbVie Limited, Dublin, Ireland) using an echocardiographic system (General Electrics, Milwaukee, WI, USA) equipped with a 10-MHz linear-array transducer, as previously reported [24]. Measurements were taken at baseline and after 6 weeks of doxorubicin treatment. M-Mode and two-dimensional (2D) echocardiography was performed at the level of the papillary muscles in the parasternal short axis view. Functional parameters over five consecutive cardiac cycles were calculated using standard methods [26]. LV dimensions in end diastole (LVDd) and end systole (LVDs), anterior and posterior wall (AW and PW) thickness in diastole and systole, end-diastolic area (EDA) and end-systolic area (ESA) were measured. Fractional area change (FAC) was calculated as FAC = [(EDA–ESA)/EDA] × 100. Fractional shortening (FS) was calculated as FS = [(LVDd-LVDs)/LVDd] × 100.

#### 2.17.4. Measurement of Fibrosis

Rats were euthanized and perfused with 2% paraformaldehyde. Hearts were extracted, dehydrated, embedded in paraffin, sectioned at 5-μm thickness and mounted on glass slides. Fibrosis in the myocardium was assessed using Masson’s trichrome and Sirius red. For Masson’s trichrome staining, glass slides were incubated in Bouin’s solution for 15 min at 56 °C, washed with running water, incubated with ferric hematoxylin for 5 min, washed again and incubated with Biebrich scarlet-acid fuchsin. Shortly after, fuchsin was removed by gentle washing, and the slides were incubated with phosphomolybdic acid for 15 min, followed by anillin blue for 1 min. Finally, slides were incubated with 1% glacial acetic acid, washed with 70, 96 and 100% ethanol, incubated in xylol and mounted. For Sirius red staining, glass slides were incubated with Sirius red solution made of 4% picric acid and 0.1% Direct Red 80 in distilled water for 30 min, followed by two washes with acetic water, two washes with 100% ethanol, two washes with xylol and mounting. All staining chemicals and related reagents were from Sigma-Aldrich. Slides were observed using a Leica DM6000 inverted optical microscope (Leica Microsystems GmbH, Wetzlar, Germany) and the images were analyzed with ImageJ software (NIH). The fibrotic zone was determined by computer planimetry (Image Proplus v7.1; Media Cybernetics, Silver Spring, MD, USA). Fibrosis was expressed as a percentage of the total LV area and as a mean of all slices from each heart.

#### 2.17.5. Immunofluorescence

For immunofluorescence staining 5-μm-thick heart slices were blocked and permeabilized with a blocking and permeabilization solution made of 5% FBS and 0.1% Triton-X100 (Sigma-Aldrich) in PBS for 1 h at RT and stained with 1/200 polyclonal rabbit IgG to caveolin-1 (#3238S; Cell Signaling Technology, Danvers, MA), 1/100 monoclonal mouse IgG anti-4 hydroxynonenal antibody (ab48506; Abcam, Cambridge, UK) or 1/400 anti-periostin (Proteintech, Manchester, UK), in the same solution overnight at 4 °C. Heart slices were then washed and stained with an Alexa Fluor^®^ 647 goat anti-rabbit IgG secondary antibody (Invitrogen, Carlsbad, CA, USA) or Alexa Fluor^®^ 488 goat anti-mouse IgG (Invitrogen, Carlsbad, CA, USA), respectively for 2 h at RT in the dark. Heart sections were washed and counterstained with 200 ng/mL DAPI (ThermoFisher Scientific) for 10 min in the dark. The heart slices were finally washed and mounted with FluorSave^TM^ reagent (CalbioChem, San Diego, CA, USA). Slides were imaged with a Leica DM2500 fluorescence microscope and analyzed with ImageJ software.

#### 2.17.6. TUNEL

For cardiomyocyte apoptosis quantification, we performed a TUNEL staining according to manufacturer’s instructions. Briefly, 5-μm-thick heart slices were blocked and permeabilized with 5% FBS and 0,1% Triton-X100 in PBS for 1 h and then stained with the TUNEL reaction mix (In situ cell death detection kit, TMR red, Roche, Basel, Switzerland) for 1 h at 37 °C in the dark. Heart sections were washed and counterstained with 200 ng/mL DAPI for 10 min in the dark. Finally, the heart slides were washed and mounted with FluorSave^TM^ reagent. Slides were imaged with a Leica DM2500 fluorescence microscope and analyzed with ImageJ software.

#### 2.17.7. Measurement of Cardiomyocyte Cross-Sectional Area in Cardiac Tissue by WGA Staining

For cardiomyocyte cross-sectional area measurement, we performed an WGA staining according to manufacturer’s instructions. Briefly, 5-μm-thick heart slices were stained with 5 μg/mL WGA (wheat germ agglutinin, Texas Red^TM^ conjugate, ThemoFisher Scientific) in HBSS for 1 h at RT. Heart slices were then washed and counterstained with 200 ng/mL DAPI for 10 min in the dark. The heart slices were finally washed and mounted with FluorSave^TM^ reagent. Slides were imaged with a Leica DM2500 fluorescence microscope and analyzed with ImageJ software.

### 2.18. Gene Ontology Analysis of Putative miR-4732-3p Target Genes

Enriched gene ontology terms for miR-4732-3p targets were retrieved from miRWalk [27] and considered significant for *p*-values < 0.05. Selected GO gene sets and their intersections were represented with an Upset plot. Using mirDIP 4.1 [28], we retrieved a large set of predicted miR-4732-3p target genes (top 33% scores, M set) that we analyzed with GeneTrail [29] to identify miRNAs with similar targeting. miRNAs that had significantly enriched validated targets were selected for downstream interactome analysis.

### 2.19. Interactome Analysis

Functional blocks from the gene ontology (GO) biological processes were used in the analysis with Cytoscape software v3.7.2 (www.cytoscape.org/, accessed on 15 April 2022). Interactome images were constructed using miRNAs detected in miRTarBase. A significance level of 0.05 was set, and functional blocks were considered enriched if their corrected *p*-value was < 0.05.

### 2.20. Statistical Analysis

Quantitative variables are presented as mean (standard deviation, SD). Analyses were conducted with SPSS software 20.0 (SPSS Inc., Chicago, IL, USA) and GraphPad Prism 6. For patient data, quantitative variables are presented as mean (SD) and categorical variables are presented as absolute and relative frequencies (number of patients and percentage). The normality of the distribution was analyzed with the Kolmogorov-Smirnov test. To compare means in the variables with normal distribution, Student’s t test was used for paired samples for intragroup comparisons and for unpaired samples in the comparison between groups. When the distribution was not normal, the Mann-Whitney U-test was used to compare two means, and the Chi-square test to compare proportions. A *p*-value < 0.05 was considered significant. The Mann-Whitney test was also used for comparison of cases versus controls in in vitro and in vivo experiments, and Student’s *t*-test was used for in vitro studies with cardiomyocytes cultures.

For RNAseq studies, as the analysis of RNAseq data can be very sensitive to the modeling decisions taken at each step, we employed three different methods, namely, the Robinson and Smyth exact negative binomial test [30], random forest [31] and elastic net [32]. We searched for miRNAs that could be used to discriminate between cases (patients that developed early-onset chronic cardiotoxicity one year after completion of chemotherapy) and controls (patients matched in terms of cancer subtype but with no loss of cardiac function) after anthracycline treatment. The results of these analyses produced a list of miRNAs dysregulated in cases versus controls. Volcano plots were used to represent the differential expression of miRNAs in serum samples from the main cohort, with the degree of significance represented as the negative logarithm of their *p*-values and the magnitude of the difference as the Log_2_ fold-change (FC). The Dseq2 method from the R package Dseq2 was used for shrinkage estimation of dispersions and fold-changes, to improve the stability and interpretability of estimates [33]. All statistical analyses were performed using R (version 3.6.1) and R-packages random forest (version 4.6-12), glmnet (version 2.0–16), NBPSeq (version 0.3.0), lme4 (version 1.1-19) and rms (version 5.1-2) (R Core Team. R Foundation for Statistical Computing, Vienna, Austria).

## 3. Results

### 3.1. Characterization of the Patient Cohorts

We prospectively followed two cohorts of patients with breast cancer receiving anthracycline chemotherapy: the main cohort comprised 20 patients from HuiPLaFe, and 32 patients from HCSC formed the validation cohort. The study was conducted as a case–control observational study. Patients were subjected to different anthracycline-based chemotherapeutic schedules chosen by the treating oncologist (Table 1).

Cardiac function was evaluated at different time points before (Pre), after chemotherapy (Post), and up to one year after completion of chemotherapy (Rev). Patients that exhibited cardiotoxicity, defined as an endpoint decline in LVEF during the first year after treatment, were classified as cases, whereas the remainder formed the control group (Table 2).

No significant differences were observed between case and control groups in either cohort regarding clinical characteristics, cardiovascular risk factors and cancer subtype (Table 1).

A significant reduction in LVEF was observed after anthracycline treatment, reaching pathological levels, in cases from both cohorts when compared with controls. No significant differences were observed for serum biomarkers between cases and controls; however, there was a significant increase in ultrasensitive troponin T levels immediately after anthracycline treatment both in cases and controls, indicative of transient myocardial damage due to anthracycline infusion, which returned to basal levels in the following days (Table 2). Serum samples from 10 patients characterized as cases and 10 matched controls were obtained after chemotherapy (post) in the main cohort. In the validation cohort, plasma samples were collected from 25 controls and 7 cases.

### 3.2. Assessment of Discriminating miRNAs between Cases and Controls

Serum samples taken after chemotherapy (Post) from patients of the main cohort were analyzed by RNAseq on the Illumina platform. From the analysis of the 2546 miRNAs in miRBase, 212 serum miRNAs were detected and normalized by relative abundance. The dataset is available in the BioStudies database (http://www.ebi.ac.uk/biostudies accessed on 2 June 2020) under accession number S-HECA437.

**Figure 1:**
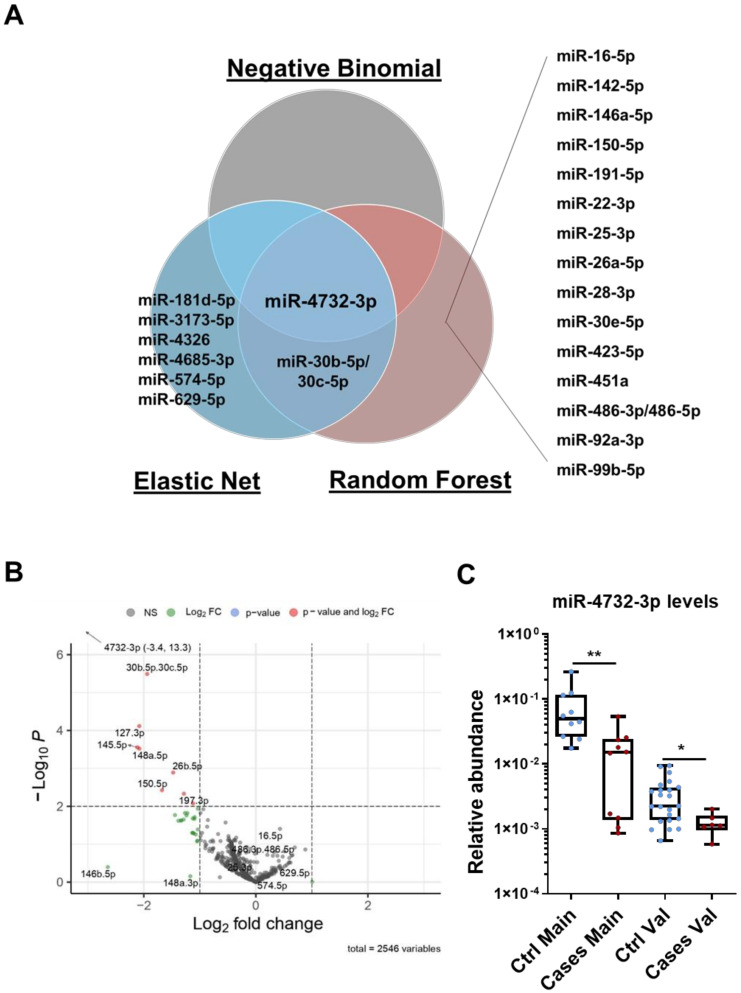
miRNA expression profiles in patients with breast cancer and early-onset cardiotoxicity. (**A**) Venn diagram of overlapping miRNAs differentially expressed in cases versus controls, after chemotherapy, from the main cohort as analyzed by negative binomial, random forest and elastic net regression methods. Data were analyzed using miRNA counts of RNAseq array. (**B**) Volcano plot after completion of chemotherapy showing individual values of 220 miRNAs detected in serum. Most significant miRNAs in relative abundance (−Log_10_ *P*) in cases versus controls are labeled in red (lower expression), blue (equal expression) and green (higher expression) as determined by Log2fold change. (**C**) miR-4732-3p levels assessed by qPCR in cases and controls from the main and the validation cohorts. Data are given as median, minimum, maximum and are shown graphically as box-and-whiskers plots for cases (red spots) and controls (blue spots). Statistical comparisons in cases versus controls after chemotherapy were performed with the non-parametric Mann-Whitney test. The experiments were performed three times. * *p* < 0.05; ** *p* < 0.01.

Using three different regression models, elastic net, negative binomial and random forest, we identified a panel of 25 miRNAs differentially expressed between cases and controls. Of these, 19 were identified by the random forest model as potentially the most important predictors for discriminating the two groups. The elastic net identified 9 miRNAs, two of them included in the panel selected by the random forest model (miR-30b-5p and miR-30c-5p) and one also selected by the negative binomial test (miR-4732-3p). The combined results of the three modeling approaches are shown in the Venn diagram in Figure 1A. The negative binomial test found significant differences only for miR-4732-3p. Indeed, this miRNA was the only one identified by all regression models. We next generated a volcano plot to visualize and compare highly dysregulated miRNAs in cases and controls. An increased proportion of miRNAs with lower abundance levels was observed in cases versus controls after anthracycline treatment, with miR-4732-3p being the most significantly downregulated (Figure 1B). Levels of miR-4732-3p were validated by qPCR in the main cohort, and differences in expression between controls and cases were confirmed. miR-4732-3p levels were also assessed in the validation cohort, which confirmed a significant difference between cases and controls (Figure 1C), although the relative abundance was lower due to the lower abundance of miRNAs in plasma.

### 3.3. miR-4732-3p Is Present in Cardiac Cells and Confers Protection against Doxorubicin Challenge

**Figure 2:**
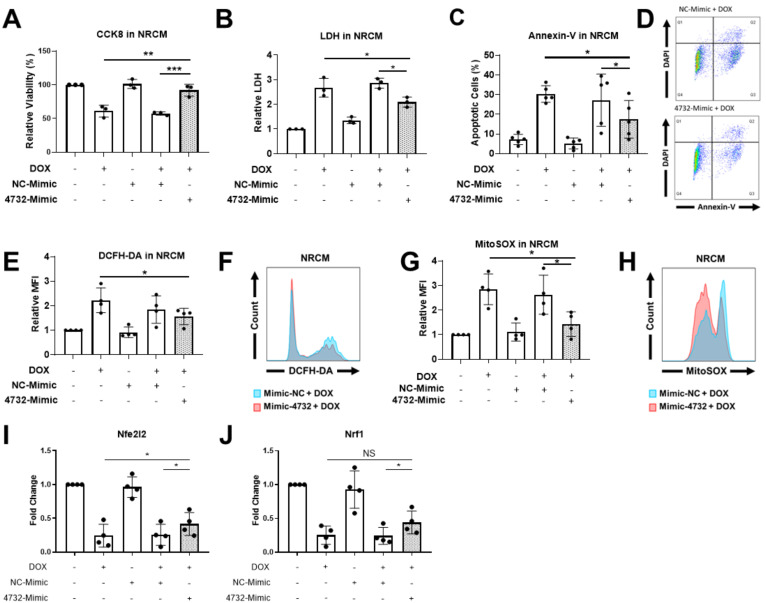
Effect of miR-4732-3p on doxorubicin-treated NCRMs. Cells were transfected with the NC-Mimic or 4732-Mimic (40 nM) followed by doxorubicin treatment (1 μM, 48 h). (**A**) Relative viability measured by CCK8 assay. (**B**) Lactate dehydrogenase activity measured in cell culture medium (**C**). Percentage of apoptotic cardiomyocytes (**D**). Representative images of flow cytometry used for apoptosis quantification showing levels of DAPI and Annexin-V. (**E**) Whole cell ROS quantification in NRCMs transfected with NC-Mimic or 4732-Mimic (40 nM) followed by doxorubicin treatment (5 μM, 24 h). Cells were stained with DCFH-DA and analyzed by flow cytometry. (**F**) Representative images of flow cytometry showing mean fluorescence intensity (MFI). (**G**) Mitochondrial superoxide quantification in NRCM treated as in (**E**) were stained with MitoSOX^TM^ and the fluorescence intensity was analyzed by flow cytometry. (**H**) Representative images of flow cytometry are shown. Data are represented as mean ± standard deviation. (**I**,**J**) *Nfe2l2* and *Nrf1* levels in NRCM treated with NC-Mimic or 4732-Mimic (40 nM) followed by doxorubicin treatment (5 μM, 24 h) * *p* < 0.05, ** *p* < 0.01, *** *p* < 0.005.

**Figure 3:**
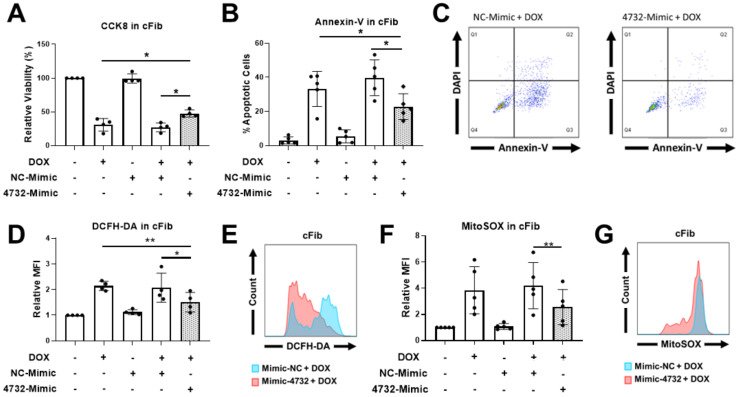
Effect of miR-4732-3p on doxorubicin-treated cFib. Cells were transfected with the NC-Mimic or 4732-Mimic (40 nM) followed by doxorubicin treatment (1 μM, 48 h). (**A**) Relative viability measured by CCK8 assay. (**B**) Percentage of apoptotic cells. (**C**) Representative images of flow cytometry used for apoptosis level quantification showing Annexin-V and DAPI in cFib. (**D**) Whole cell ROS quantification in cFib transfected with NC-Mimic or 4732-Mimic (40 nM) followed by doxorubicin treatment (5 μM, 24 h). Cells were stained with DCFH-DA and analyzed by flow cytometry. (**E**) Representative images of flow cytometry showing mean fluorescence intensity (MFI). (**F**) Mitochondrial superoxide quantification in cFib treated as in (**D**) were stained with MitoSOX^TM^ and the fluorescence intensity was analyzed by flow cytometry. (**G**) Representative images of flow cytometry are shown. Data are represented as mean ± standard deviation. * *p* < 0.05, ** *p* < 0.01.

Our previous results demonstrating the cardioprotective features of miR-4732-3p against oxygen and glucose deprivation in NRCM [21] inspired us to determine whether it could also confer protection against doxorubicin challenge in cardiac cells. We first determined the abundance of miR-4732-3p in NRCM and cFib using qPCR. The absolute number of miR-4732-3p copies per 10^5^ seeded cells was 6372 ± 1214 for NRCM and 47,009 ± 13,401 for cFib. We also wanted to assess the relative abundance of miR-4732-3p in cardiac tissue. Copy numbers of this miR were higher in cardiac tissue than in other tissues, such as liver, brain, lung and spleen (Supplemental Figure S1). We next tested whether anthracycline treatment modulated miR-4732-3p levels in cardiac cell cultures, as observed in patient blood samples, by treating NRCM and cFib with doxorubicin for 48 h. We found that the expression of miR-4732-3p was elevated after doxorubicin treatment (Supplemental Figure S2A,B). To test whether miR-4732-3p confers protection against cardiomyocyte death after anthracycline treatment, we transfected NRCM with a miR-4732-3p mimic (4732-Mimic) using Lipofectamine 3000, and measured cell viability after doxorubicin challenge. As a transfection control, we employed a negative control mimic (NC-Mimic). The 4732-Mimic transfection did not affect cell viability of NRCM at the doses assessed (Supplemental Figure S3). Results showed that the 4732-Mimic conferred a significant degree of cardioprotection after doxorubicin challenge in terms of cell viability (Figure 2A), lactate dehydrogenase release (Figure 2B) and apoptosis, measured by Annexin-V staining (Figure 2C). Likewise, oxidative stress was significantly suppressed by miR-4732-3p, as shown by lower levels of DCFH-DA and MitoSOX^®^ fluorescence in 4732-Mimic-treated cells than in doxorubicin treated cells, indicating lower levels of total ROS and mitochondrial superoxide, respectively (Figure 2E,G). We also found that miR-4732-3p increased mRNA levels of *Nfe2l2* and *Nrf1*, two transcription factors that regulate genes encoding proteins involved in response to injury involving free radicals [34,35]. Similar results were obtained in cFib (Figure 3A–G), indicating that miR-4732-3p is cardioprotective against doxorubicin challenge. To test whether miR-4732-3p also conferred protection to breast cancer cells, which would clearly limit its clinical utility, we added 4732-Mimic to two different cell lines, the highly invasive triple negative MDA breast cancer cell line and the non-metastatic MCF7 breast cancer cell line [36], and analyzed cell viability after doxorubicin treatment. Notably, the 4732-Mimic failed to protect against anthracycline treatment as shown by no changes to cell viability when compared with the negative control (Supplemental Figure S4). Overall, these findings suggest that miR-4732-3p might have utility as a cardioprotective agent in anthracycline-based therapies without diminishing its anti-tumor effects.

### 3.4. miR-4732-3p Administration Preserves Cardiac Function after Doxorubicin Challenge

**Figure 4:**
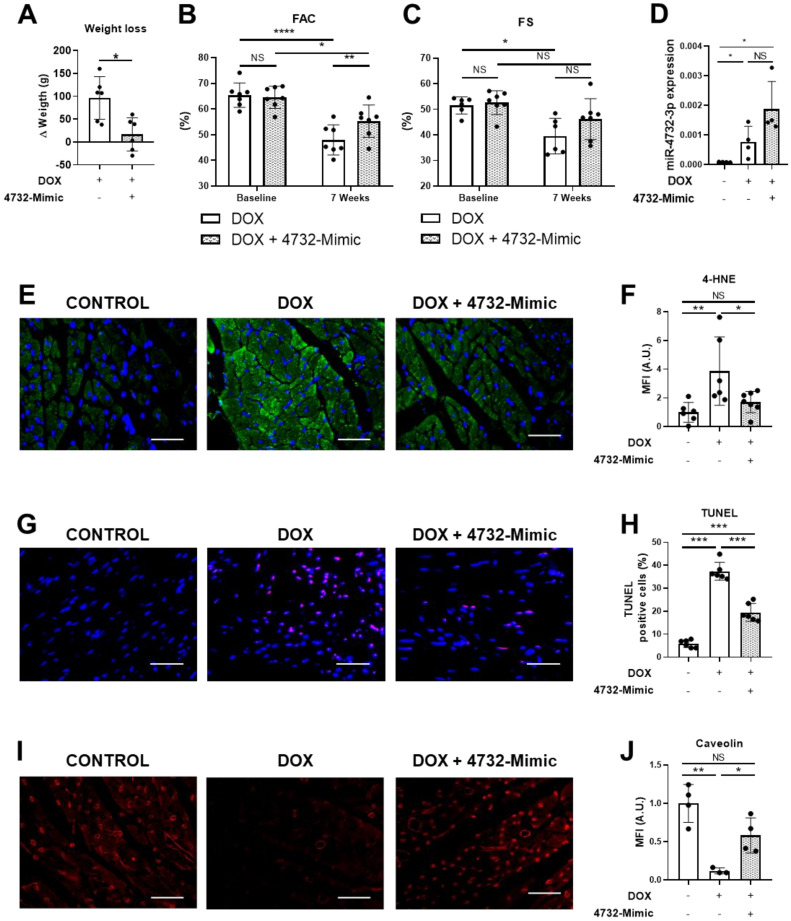
Effect of miR-4732-3p on doxorubicin-treated rats. (**A**) Weight loss in doxorubicin-treated animals after i.v. injection of saline or 4732-Mimic (*n* = 6). (**B**) Prevention of left ventricle function decline in 4732-Mimic-treated animals. Quantified values of area fractional change (FAC) are shown (*n* = 7). Data are expressed as mean ± SEM. (**C**) Effect of 4732-Mimic on fractional shortening (FS) (*n* = 7). (**D**) Plasma levels of miR-4732-3p. (**E**) Representative images of anti-HNE-stained heart sections (scale bar = 100 μm). (**F**) Quantification of 4-HNE positive area in the heart sections (*n* = 6) (**G**) Representative images of TUNEL-stained heart sections (scale bar = 100 μm.) (**H**) Quantifications of TUNEL positive cells expressed in percentage of apoptotic cells per total cardiac cells (*n* = 6) (**I**) Representative images of anti-caveolin-stained heart sections (scale bar = 100 µm). (**J**) Quantification of caveolin-positive area in the heart sections (*n* = 5). * *p* < 0.05, ** *p* < 0.01, *** *p* < 0.005, **** *p* < 0.001.

We next tested whether the administration of miR-4732-3p could prevent loss of cardiac function in rats after prolonged doxorubicin treatment. Results were compared with animals administered with saline. Rats were treated weekly with doxorubicin for 6 weeks. The 4732-Mimic was injected in the first and third week of treatment. Six weeks after the initiation of doxorubicin treatment, cardiac function parameters were measured to assess the degree of damage. Animals treated with the 4732-Mimic experienced less weight loss than the control group (Figure 4A). The 4732-Mimic group also exhibited significant preservation of systolic function as calculated by the FAC percentage (Figure 4B). Fractional shortening (FS) was not significantly improved by miR-4732-3p administration, although we observed a tendency towards cardioprotection (Figure 4C). Furthermore, our results showed that miR-4732-3p was upregulated in plasma samples of rats treated with doxorubicin and doxorubicin with 4732-Mimic compared to control ones (Figure 4D). To test whether miR-4732-3p could protect from oxidative stress in vivo, we performed an immunofluorescence staining against 4-hydroxynonenal (4-HNE), which is a marker of lipid peroxidation. We observed that animals treated with 4732-Mimic showed significantly less 4-HNE staining than rats treated with doxorubicin alone (4E, F). Furthermore, we wanted to see whether miR-4732-3p could prevent apoptosis in vivo, so we performed a TUNEL assay, which labels nuclei of cells undergoing apoptosis. Treatment with doxorubicin increased levels of apoptosis compared to control. However, the treatment with 4732-Mimic during doxorubicin challenge reduced the number of apoptotic cells compared with animals treated only with doxorubicin (Figure 4G,H). Finally, a significant increase in vascularization was observed in animals treated with 4732-Mimic, as measured by blood vessel density (caveolin staining) (Figure 4I,J). A similar angiogenic effect of this mimic was previously observed in response to cardiac ischemia [21].

### 3.5. miRNA-4732-3p Reduces Fibrosis after Doxorubicin Challenge in Rats

We also evaluated whether miR-4732-3p could also reduce the fibrous scarring evident in doxorubicin-treated animals.

**Figure 5:**
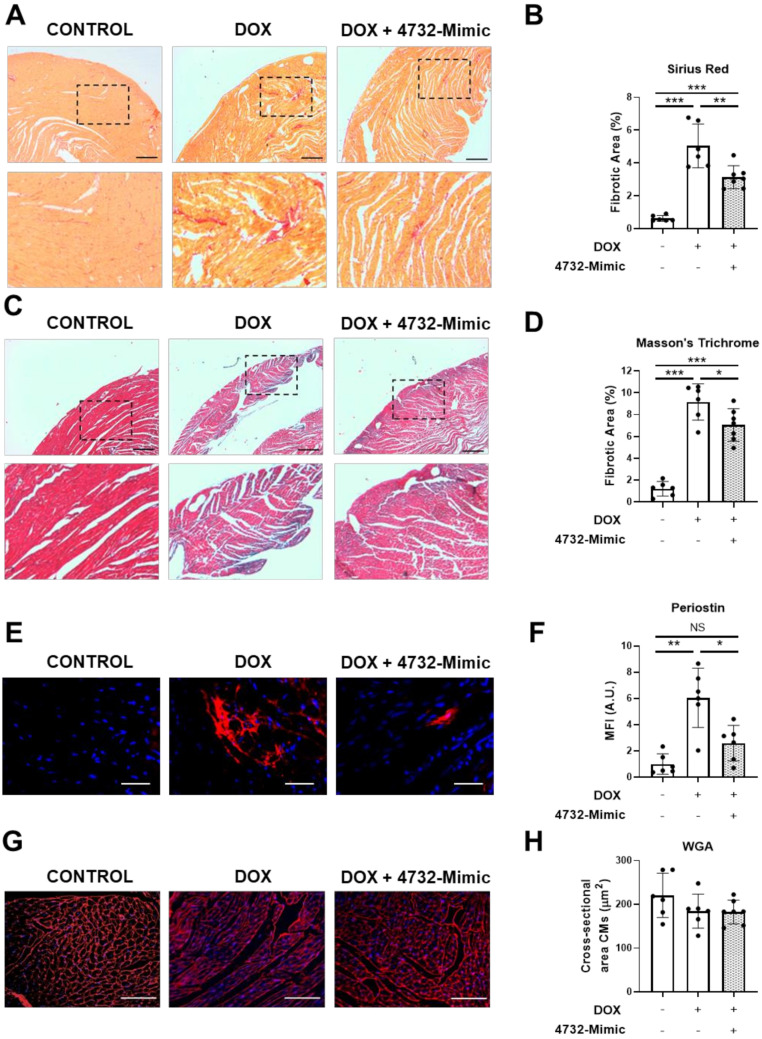
Effect of 4732-3p mimic on fibrosis and hypertrophy in doxorubicin-treated rats. (**A**) Representative heart sections stained with Masson’s trichrome from doxorubicin-treated rats with or without i.v. injection of 4732-Mimic (scale bar = 500 μm). (**B**) Fibrotic area in Masson’s trichrome-stained sections was calculated as a percentage of blue area in total section area. (**C**) Representative heart sections stained with Sirius red from doxorubicin-treated rats with or without i.v. injection of 4732-Mimic (scale bar = 500 μm). (**D**) Fibrotic area in Sirius red-stained sections was calculated as a percentage of intense red area in total section area. (**E**) Representative images of anti-periostin-stained heart sections (scale bar = 100μm). (**F**) Quantification of periostin-positive area in the heart sections (*n* = 6). (**G**) Representative heart sections stained with WGA (scale bar = 100μm). (**H**) Quantification of cross-sectional area of cardiomyocytes. Animals were euthanized 4 weeks after the last infusion of 4732-Mimic. Values are the mean ± SEM corresponding to *n* = 6 and *n* = 7 for control and 4732-Mimic groups, respectively. * *p* < 0.05, ** *p* < 0.01, *** *p* < 0.005.

Sections of heart tissue from 4732-Mimic and control groups (Section 3.4) were analyzed in animals euthanized 6 weeks after the initiation of doxorubicin. Sections were stained with Masson´s trichrome or Sirius red (Figure 5A,C). In both cases, the 4732-Mimic group showed lower staining (measured as fibrotic area) compared with the saline group (Figure 5B,D). Since the major event to trigger fibrotic processes is the activation of fibroblasts to differentiate into myofibroblasts and promote extracellular matrix deposition, we stained heart sections with antibodies against periostin, a well-known marker of myofibroblasts [37,38]. As expected, doxorubicin treatment increased the number of periostin-positive cells in the heart. However, the intramyocardial injection of 4732-Mimic induced a patent reduction of this process, and a scarce number of myofibroblasts was observed in treated animals (Figure 5E,F). Lastly, it has been described that some microRNAs have a role in regulation of cardiac hypertrophy [39]. Thus, we aimed to evaluate whether 4732-Mimic could induce cardiac hypertrophy. The cardiomyocyte area in the border zone of the infarct was calculated from heart sections stained with anti-WGA antibodies (Figure 5G,H). Analysis of cardiomyocyte cross-sectional area, quantified by WGA staining, revealed no differences in the two groups, indicating the absence of cardiac hypertrophy after 4732-Mimic administration. Overall, these in vivo data support the hypothesis that miR-4732-3p induces cardioprotection against doxorubicin challenge.

### 3.6. Identification and Validation of Putative miR-4732-3p Target Genes

**Figure 6:**
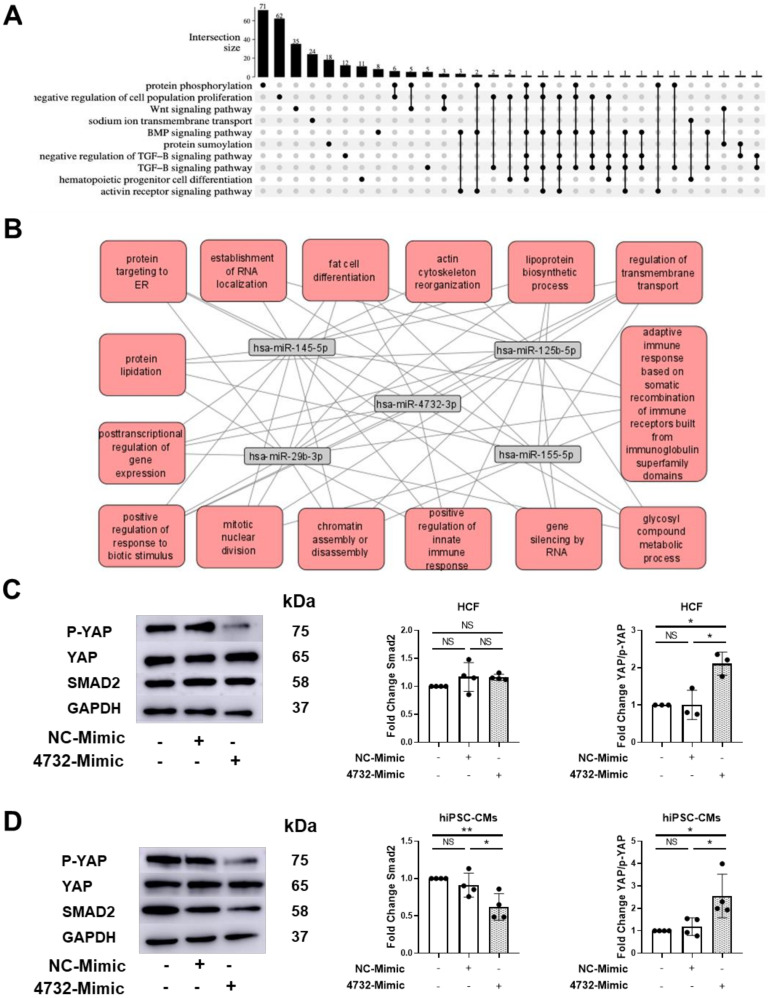
Identification and validation of miR-4732-3p target genes. (**A**) GO terms among predicted targets of miR-4732-3p and their intersections. (**B**) Interactome of combined target genes of miR-4732-3p, miR-145-5p, miR-125b-5p, miR-29b-3p, miR-155-5p. (**C**) Representative western blots of p-YAP, YAP and Smad2 in HCF non-transfected or transfected with either NC-Mimic or 4732-Mimic. Relative levels were quantified by densitometry relative to the levels of HCF without transfection. GAPDH was used as a loading control. (**D**) Representative western blots of p-YAP, YAP and Smad2 in hiPSC-CMs non-transfected or transfected with either NC-Mimic or 4732-Mimic. Relative levels were quantified by densitometry relative to the levels of hiPSC-CMs without transfection. GAPDH was used as a loading control. Data are represented as mean ± standard deviation. * *p* < 0.05, ** *p* < 0.01.

To gain some insights into the mechanism of action of this miRNA at the molecular level, we decided to explore in silico the miR-4732-3p target genes and biological processes (BP) regulated by this molecule. First, we retrieved from miRWalk significantly enriched Gene Ontology (GO) terms among predicted targets [27]. Selected sets of genes (Supplemental Table S1) and their intersections were represented using an UpSet plot as overview of the most relevant GO terms related to miR-4732-3p (Figure 6A). We observed considerable overlaps among targets belonging to transforming growth factor beta (TGF-β) signaling pathway, which made it an interesting candidate for downstream validation. Using Gene Trail, we identified miR-29b-3p, miR-125-5p, miR-145-5p, miR-155-5p as miRNA that showed common targets with miR-4732-3p (Supplemental Table S2). As miRNA often act in concert, we used the combined targets of the five miRNA and performed an interactome analysis to visualize common target genes and BP in which they were involved (Figure 6B and Supplemental Table S3). The overlapping canonical pathways were mapped using Cytoscape to allow for visualization of the shared biological pathways through the common genes. Unfortunately, the nodes identified pointed to quite broad BP that could not be easily related to the cardioprotective effect of miR-4732-3p observed in vitro and in vivo, so we decided to investigate gene targets belonging to the TGF-β and Hippo signaling pathways that could shed light on the regenerative capacity of this miRNA. For this purpose, we validated the previously identified target SMAD2 [40], together with yes-associated-protein 1 (YAP) and its phosphorylated form (p-YAP) as effectors of non-canonical Wnt and Hippo signaling pathways that play a pivotal role in cardiac regeneration [41,42,43] (Figure 6C,D and Supplemental Figure S5A). The validation and identification of new target genes was performed in the human kidney HEK 293 cell line as a non-cardiac cell line and in human cardiac fibroblasts (HCF) and cardiomyocytes (hiPSC-CM) as cardiac cell lines. Thus, we transduced HEK 293 with a miR-4732-3p lentiviral vector or transfected HCF and hiPSC-CM with lipofectamine and the 4732-mimic or NC-mimic. In all the cell lines we observed a marked overexpression of miR-4732-3p after infection or transfection of mimic miRNA using lipofectamine (Supplemental Figure S5B–D). miR-4732-3p mediated modulation of SMAD2 was only observed in iPSC-CMs and HEK 293 (Figure 6C,D and Supplemental Figure S5A), whereas the modulation of phospho-YAP was observed in the three cell types after miR-4732-3p overexpression (Figure 6C,D and Supplemental Figure S5A), indicating that YAP is not a target itself of miR-4732-3p, but this miRNA is able to modulate its activations by reducing the levels of its phosphorylated form.

## 4. Discussion

Cardiotoxicity is a major collateral effect in breast cancer patients treated with anthracyclines [6]. Few biomarkers are currently available to identify breast cancer survivors at risk of heart deterioration. Here, we investigated the associations between miRNAs, identified by RNAseq and regression analysis, and diminished cardiac function measured by echocardiography in this patient group. Using this approach, we identified 25 microRNAs associated with early cardiotoxicity after anthracycline treatment, with potential as biomarkers or therapeutic products to prevent/treat cardiotoxicity. Supporting this miRNA signature, several studies have associated some of these miRNAs with cardiomyocyte damage after doxorubicin treatment. For example, *Rattus norvegicus* rno-miR26, rno-miR-30, rno-146a and rno-miR-150, homologous to the human counterparts, were reported to be dysregulated after doxorubicin treatment in adult rat ventricular myocytes and in an in vivo rat model of doxorubicin-induced chronic heart failure [44]. Moreover, in a recent systematic review, miR-16-5p, miR-25-3p, miR-92a and miR-486 were associated with anthracyclines-induced cardiotoxicity in breast cancer patients [45]. Likewise, miR-486-3p and miR-486-5p showed differential doxorubicin-responsive expression before the occurrence of other cytotoxicity markers (such as lactate dehydrogenase changes) and have been demonstrated to have a significant involvement in heart failure in patients and cellular models [9]. It is worthy to mention that miR-1 was previously described as a circulating biomarker in breast cancer patients that experienced cardiotoxicity [46]. This microRNA was not detected as differentially expressed in our study. A plausible explanation is that the authors detected this biomarker in plasma samples while we used serum samples, and often levels of microRNAs are not comparable between both types of fluids.

We focused on miR-4732-3p, the most dysregulated microRNA in patients experiencing cardiotoxicity in our study, as assessed by three different regression models. qPCR analysis in two independent cohorts confirmed that this miRNA was downregulated in cases versus controls. We also observed that the levels of miR-4732-3p were dramatically lower in plasma samples than in serum samples. Although little is known about the origin of miR-4732-3p, it is encoded within the erythroid-enriched miR-144/451 locus, and reductions in the level of this miRNA are related to reduced erythroid cell survival and limited erythropoiesis [40], supporting its erythroid origin. Nonetheless, miR-4732-3p is also present in cells of solid organs, as demonstrated here. Of note, miR-4732-3p is part of an miRNA signature of heart failure [47], indicating its possible involvement in cardiac pathophysiological processes. To better understand the mechanism underlying the regulatory role of miR-4732-3p, we administered a synthetic mimic of this mRNA in cardiac cells and subjected cultures to doxorubicin treatment. The mimic was encapsulated in liposomes, which allowed delivery into cells and tissues. Transduction of NRCM and cFib with the 4732-Mimic alleviated oxidative stress and apoptosis in both cell types and preserved cell viability. Consistently, the 4732-Mimic infused intravenously in rats during doxorubicin treatment preserved cardiac function and decreased oxidative stress and apoptosis. Additionally, 4732-Mimic reduced fibrosis and myofibroblasts accumulation in myocardial tissue, likely due to prevention of fibroblast differentiation into myofibroblasts and to an angiogenic response, in the absence of cardiac hypertrophy. Indeed, anti-fibrotic therapy targeting myofibroblasts has been proposed for the treatment of patients with end-stage heart failure [38]. This finding is in good agreement with our previous study showing that miR-4732-3p can protect the heart against hypoxia in a rat model of myocardial infarction by inducing angiogenesis and reducing infarct size [21]. The fact that miR-4732-3p can exert cardioprotective effects against two different types of myocardial damage points to its involvement in the anti-apoptotic and anti-oxidative mechanisms essential for cardiac tissue repair.

We also explored the regulation of Smad2, a component of TGFβ pathway, and YAP and phospho-YAP, as major effectors of Hippo signaling [41,42,43], in HEK 293 cells and human cardiac cells (HFB and iPSC-CMs). It has been reported that miR-4732-3p promotes proliferation by suppressing Smad2 and Smad4 in human CD34+ erythroid progenitors [40]. We observed that overexpression of miR-4732-3p resulted in a reduction of Smad2 levels in iPSC-CM and HEK 293. However, in our experimental conditions, we did not observe changes in Smad2 levels by miR-4732-3p overexpression in HCF, which could indicate a different role of this miRNA, depending on the cell type. Regarding YAP, the downstream coactivator of Hippo pathway, the results presented here are preliminary, and the identification of the miR-4732-3p target gene involved in YAP phosphorylation remains to be determined. Nonetheless, the observation that miR-4732-3p regulates phospho-YAP levels in all cell types tested is interesting, since reduction in phosphorylation could prevent cytoplasmic sequestration of YAP, promoting the nuclear translocation of this transcription factor and the activation of the Hippo pathway [48]. In this context, it was recently described that phospho-YAP levels were increased in cardiac cultures treated with doxorubicin, but the cardioprotective drug Melatonin was able to decrease them in comparison to non-treated cultures with this drug. Additionally, the authors demonstrated that YAP levels contributed to the cardioprotective effect of Melatonin in doxorubicin-induced cardiotoxicity both in vitro and in vivo since YAP-siRNA abolished this effect [49]. Moreover, the fact that this effect was observed, regardless of the cell type we tested, allows us to speculate that miR-4732-3p plays a pro-survival role, not only in the context of cardiotoxicity and heart failure, but also in other stress conditions. Indeed, this microRNA was the only one dysregulated after low-Earth orbit space flights in astronauts´ plasma-derived small extracellular vesicles cargo, and in silico studies correlated it with brain and heart processes [50]. This is also in line with our functional analysis of miR-4732-3p predicted targets which include genes from the TGFβ, BMP and VEGF pathways that exert pivotal roles in survival processes such as cardiac repair and regeneration (Figure 6A and Supplemental Table S2) [51,52]. In this context, the in silico analysis showed a strong evidence of interaction with miR-29b-3p, miR-125b-5p, miR-145-5p and miR-155-5p. Although the interactome studies did not provide specific pathways in which these molecules are involved, previous reports demonstrated their role in similar processes. For example, miR-29 is downregulated after myocardial infarction, and its inhibition induces cardiac fibrosis [53]. Downregulation of miR-29a-3p was associated to TGF-β mediated pulmonary fibrosis, and this miRNA showed a key role in hypoxia-induced activation of pulmonary adventitial fibroblasts [54]. miR-125-5p was involved in the control of inflammatory response and cell apoptosis and associated with ischemic stroke [55,56]. miR-145 was downregulated in patients with heart failure and has been related to TGFbeta and Wnt/beta catenin signaling pathways [57]. miR-155-5p promoted interstitial fibrosis and inhibition of miR-155-5p improved cardiac function following myocardial infarction in mice, by decreasing cardiomyocyte apoptosis and increasing angiogenesis in infarcted hearts [58]. Interestingly, miR-155-5p was up-regulated after isoproterenol induced cardiotoxicity [59]. However, to our knowledge, no relationship between the other microRNA and cardiotoxicity has been reported.

Other miRNAs have been described with similar cardioprotective features against anthracyclines and ischemia, but none of them have been tested against both types of cardiac insult. For example, miR-148a targets gp-130, a co-receptor for cytokines of the cytokine IL-6 superfamily, and its delivery using adeno-associated vectors prevents cardiac dilation and dysfunction in mice induced by experimental pressure-overload [60]. By contrast, the transgenic overexpression of miR-212/132 in mice is known to induce cardiac hypertrophy, and administration of an antisense oligonucleotide inhibitor prevented this in various models of heart disease, including a pig model of heart failure [61]. In another study, the expression of miR-19a/19b was found to be induced in patients with heart failure, and intracardiac injection of synthetic mimics of these molecules elicited cardioprotective responses in mice, both at early stages after acute myocardial infarction and long-term [62]. As mentioned earlier, in the context of cardioprotection against doxorubicin challenge, miR-30 was identified to be significantly downregulated after doxorubicin treatment in rats, whereas its overexpression was protective and reduced oxidative damage after doxorubicin challenge by targeting BNIP3L/NIX [44].

The present study has several limitations. First, the number of cases in the main and validation cohort was low, so further verification with larger cohorts will be needed. Second, the anti-apoptotic, antioxidant and pro-survival responses triggered by miR-4732-3p were tested in murine cells. Although it is likely that similar effects will be observed in human cells, the use of HCF and iPSC-CMs would be desirable. Third, cardiac function measurements revealed a preservation of fractional area changes in doxorubicin-treated animals in response to 4732-Mimic infusion, but no differences were observed in fractional shortening. This suggests that although myocardial tissue preservation was patent, in terms of reduced fibrosis and improved perfusion, additional doses or improved methods of administration will be required to avert cardiac dysfunction. Finally, we used a model of doxorubicin-induced cardiotoxicity in male rats, while cardiotoxicity by anthracyclines treatment affects both male and female patients. Verification of the effect of miR-4732-3p induced cardioprotection after doxorubicin challenge should be verified using female rats.

## 5. Conclusions

Our study shows that miR-4732-3p can exert cardioprotective mechanisms in cardiac cells by, at least in part, triggering angiogenic and anti-oxidative responses. When administered in doxorubicin-treated rats, the 4732-3p Mimic induced blood vessel formation, reduced diffuse fibrosis and prevented cardiac function deterioration. This miRNA regulates, at least in part, TGF-β and Hippo signaling pathways. In addition, lower levels of circulating miR-4732-3p might be indicative of early cardiotoxicity after anthracycline treatment in patients with breast cancer. Further studies are needed to evaluate the protective effects of miR-4732-3p against anthracycline-related myocardial damage.

## Figures and Tables

**Table 1:** Clinical characteristics of the study population.

	Main Cohort		*p*-Value	Validation Cohort		*p*-Value
	Controls (*n* = 10)	Cases (*n* = 10)	Controls vs. Cases	Controls (*n* = 25)	Cases (*n* = 7)	Controls vs. Cases
	Mean ± SD (Range)	Mean ± SD (Range)		Mean ± SD (Range)	Mean ± SD (Range)	
						
Age (years)	51.1 ± 9.5 (35–69)	53.2 ± 13.5 (38–70)	0.694	53.3 ± 10.2 (33–73)	46.3 ± 12.9 (34–80)	0.093
BMI	24.8 ± 3.1 (19–30)	25.6 ± 2.4 (23–30)	0.87	28.9 ± 5.4 (19–43)	30.7 ± 6.2 (21–37)	0.491
						
**Medical history**	*n* (%)	*n* (%)		*n* (%)	*n* (%)	
						
Hypertension (%)	0 (0%)	2 (20%)	0.171	6 (24%)	1 (14%)	0.551
DMT2 (%)	1 (10%)	2 (20%)	0.453	2 (8%)	0 (0%)	0.430
Dyslipidemia (%)	3(30%)	4 (40%)	0.478	3 (12%)	0 (0%)	0.325
Smoking (%)	1 (10%)	2 (20%)	0.231	7 (28%)	4 (57%)	0.173
						
**Cancer subtype**						
						
Triple Negative	2 (20%)	6 (60%)	0.068	1 (4.16%)	0 (0%)	0.591
Luminal A	1 (10%)	1 (10%)	1.000	8 (33.3%)	2 (29%)	0.864
Luminal B	6 (60%)	1 (10%)	0.019	13 (54.1%)	3 (4%)	0.669
HER2/positive	0 (0%)	2 (20%)	0.136	2 (8.33%)	2 (29%)	0.440
HER2 negative	1 (10%)	0 (0%)	0.305	1 (4.16%)	0 (0%)	0.591
						
**Cumulative dose**						
TAC (mg/m^2^)	293 ± 15	295 ± 0.57	0.779	501.5 ± 160	547.2 ± 215	0.562
AC (mg/m^2^)	242 ± 34	255.7 ± 65	0.673	481.24 ± 152	489 ± 110.4	0.903

BMI: body mass index; DMT2: Diabetes mellitus type 2; SD: standard deviation; HER2: human epidermal growth factor receptor 2; TAC: Taxotere (docetaxel), Adriamycin (doxorubicin), and Cyclophosphamide, AC: Adriamycin (doxorubicin) and Cyclophosphamide. Statistical comparisons in patients between groups. *p*-values corresponding to the comparison between controls and cases in each cohort and to the comparison between the main cohort and the validation cohort.

**Table 2:** Serum biomarkers and echocardiographic parameters of the study population.

		Main Cohort			Validation Cohort		
Biochemical		Controls (*n* = 10) Cases (*n* = 10)			Controls (*n* = 25) Cases (*n* = 7)		
Parameters		Mean ± SD			Mean ± SD		
		Pre	Post		Pre	Post	
**Cholesterol** **(mg/dL)**	**Control**	223 ± 35	221 ± 34		214 ± 26	205 ± 36	
	**Cases**	225 ± 46	216 ± 57		212 ± 22	190 ± 27	
					
**Triglycerides** **(mg/dL)**	**Control**	85 ± 24	189 ± 216		108 ± 48	131 ± 79 ^††^	
	**Cases**	85 ± 22	96 ± 25		96 ± 62	99 ± 47	
					
**Glucose** **(mg/dL)**	**Control**	96 ± 6	99 ± 20		95 ± 15	99 ± 17	
	**Cases**	113 ± 41	102 ± 40		87 ± 3	88 ± 8	
					
**Creatinine** **(mg/dL)**	**Control**	0.68 ± 0.1	0.59 ± 0.1 *		0.59 ± 0.09	0.56 ± 0.1	
	**Cases**	0.68 ± 0.1	0.56 ± 0.1 *		0.60 ± 0.12	0.6 ± 0.12	
					
**usTnT** **(ng/L)**	**Control**	3.1 ± 0.2	12.5 ± 9.6 ^‡^		4.9 ± 4.6	9.3 ± 5.8 *	
	**Cases**	4.6 ± 2.9	17.9 ± 8.6 ^†^		3.4 ± 0.68	5.3 ± 2.2	
				
**NTproBNP** **(pg/mL)**	**Control**	104.9 ± 60.8	99.4 ± 97.6		43.5 ± 36.9	79.8 ± 96.7	
	**Cases**	62.2 ± 50.7	125.5 ± 87.1 *		58.0 ± 28.5	46.4 ± 44.3	
					
**Echocardio.**		Controls (*n* = 10) Cases (*n* = 10)			Controls (*n* = 25) Cases (*n* = 7)		
**Parameters**			Mean ± SD		Mean ± SD		
		**Pre**	**Post**	**Rev**	**Pre**	**Post**	
**LVEF (%)**	**Control**	68 ± 4	65 ± 5 ^†^	63 ± 4 ^‡^	62.0 ± 5.2	61.3 ± 5.6	
	**Cases**	65 ± 5	57 ± 1 ^‡#^	49 ± 1 ^‡#^	63.0 ± 6.9	53.0 ± 4.0 ^†#^	
							
**LVEDV (mL)**	**Control**	94 ± 2	84 ± 2	86 ± 2	92.3 ± 10.1	89.0 ± 9.9	
	**Cases**	90 ± 4	92 ± 3	92 ± 4	106 ± 37	108 ± 28 ^||^	
							
**LVESV (mL)**	**Control**	21 ± 6	20 ± 6	21 ± 2	34.5 ± 6.7	34.3 ± 6.8	
	**Cases**	23 ± 1	23 ± 1 ^||^	26 ± 12 ^#^	42 ± 21	52 ± 18 ^†#^	
**GLS (%)**	**Control**	−17.3 ± 1.5	−16.1 ± 2.8	−17.2 ± 1.7	−22.4 ± 1.7	−21.7 ± 1.6	
	**Cases**	−16.8 ± 2.7	−15.5 ± 2.2	−12.9 ± 1.4 *^§^	−21.0 ± 1.5	−19 ± 2.5 *^§^	

LVEF: left ventricular ejection fraction; LVEDV: left ventricular end diastolic volume. LVESV: left ventricular end systolic volume. GLS: global left ventricular longitudinal strain. Mean values and standard deviation is shown in controls and cases with early-onset of cardiotoxicity at baseline (Pre) and after anthracyclines (Post) and revision (Rev). Statistical comparisons in patients at different timepoints and between groups. * *p* < 0.05; ^†^ *p* < 0.01 ^‡^ *p* < 0.001 different timepoints intragroup comparisons vs. pre; ^§^ *p* < 0.05; ^||^ *p* < 0.01; ^#^ *p* < 0.001 in cases vs. controls at each timepoint.

## Data Availability

The dataset is available in the BioStudies database (http://www.ebi.ac.uk/biostudies accessed on 2 June 2020) under accession number S-HECA437.

## Supplementary Materials

The following supporting information can be downloaded at: https://www.mdpi.com/article/10.3390/antiox11101955/s1. Figure S1: Expression of miR-4732-3p on different tissues from adult Wistar rats without treatment; Figure S2: Relative copy number of miR-4732-3p; Figure S3: Effect of 4732-Mimic transfection on NRCM; Figure S4: Effect of miR-4732-3p in breast cancer cell lines; Figure S5: Identification and validation of miR-4732-3p target genes; Table S1: significantly enriched Gene Ontology (GO) terms among predicted targets; Table S2: common targets of miR-29b-3p, miR-125-5p, miR-145-5p, miR-155-5p and miR-4732-3p; Table S3: biological processes of the common targets of miR-29b-3p, miR-125-5p, miR-145-5p, miR-155-5p and miR-4732-3p.
