# Supplementary material for: MicroRNA-4732-3p Is Dysregulated in Breast Cancer Patients with Cardiotoxicity, and Its Therapeutic Delivery Protects the Heart from Doxorubicin-Induced Oxidative Stress in Rats
Source: Antioxidants (Basel). 2022 Sep 30;11(10):1955. doi: 10.3390/antiox11101955 (PMC9599023; doi:10.3390/antiox11101955)
Supplement: Supplementary file 1 [file antioxidants-11-01955-s001.zip › antioxidants-1900564-figures.pdf]

Supplementary Materials

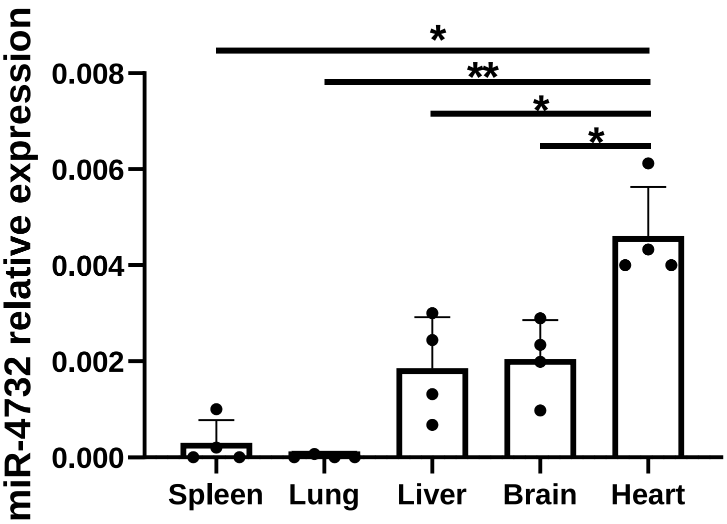

Figure S1. Expression of miR-4732-3p on different tissues from adult Wistar rats without treatment. Levels of miR-4732-3p have been relativized to levels of the small nuclear RNA U6. Data are represented as mean ± standard deviation. *p<0.05, **p<0.005.

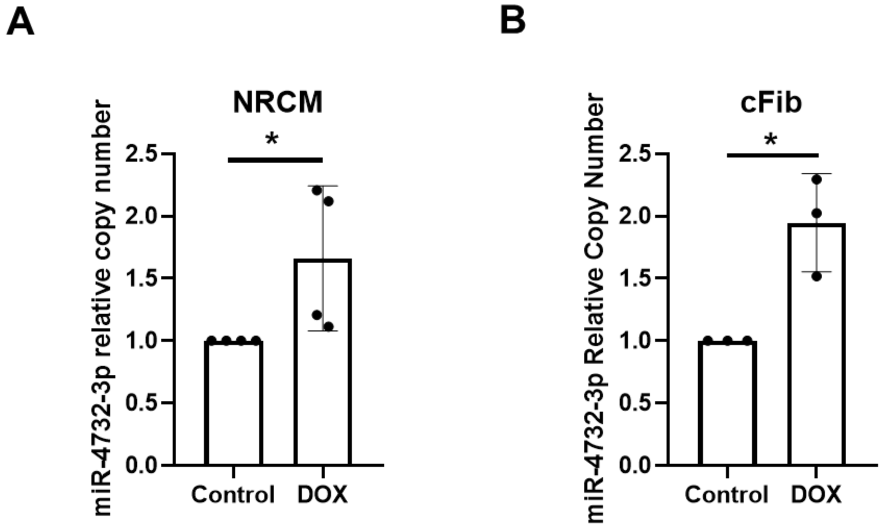

Figure S2. Relative copy number of miR-4732-3p. (**A**) Relative copy number of miR-4732-3p in NRCMs after doxorubicin treatment (1 µM, 48 h). (**B**) Relative copy number of miR-4732-3p in cFib after doxorubicin treatment (1 µM 48 h). Data are represented as mean ± standard deviation. *p<0.05.

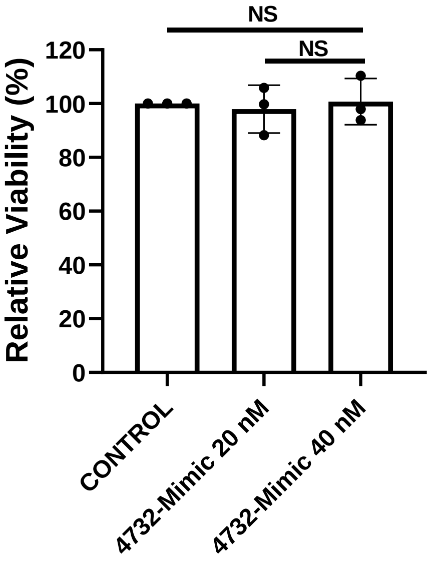

Figure S3. Effect of 4732-Mimic transfection on NRCM. NRCM were transfected with 4732-Mimic for 24 h at two different doses (20 and 40 nM) and cell viability was assessed by CCK8. Data are represented as mean ± standard deviation. *NS* = not significant.

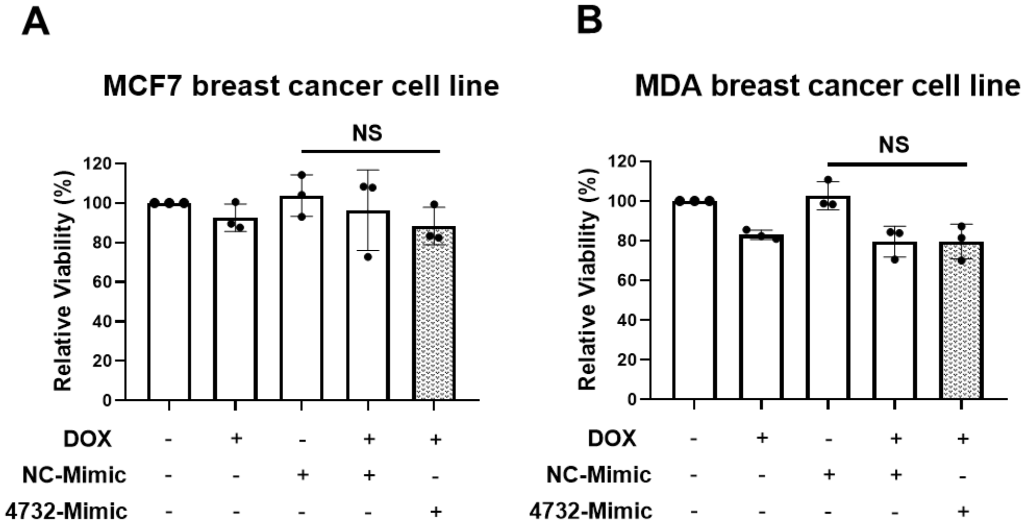

Figure S4. Effect of miR-4732-3p administration in breast cancer cell lines. (**A**) Relative viability of MCF7 breast cancer cells after NC-Mimic or 4732-Mimic transfection (40 nM) and doxorubicin treatment (1 μM, 48 h). (**B**) Relative viability of MDA breast cancer cells after NC-Mimic or 4732-Mimic transfection (40 nM) and doxorubicin treatment (1 μM, 48 h). Data are represented as mean ± standard deviation. *NS* = not significant.

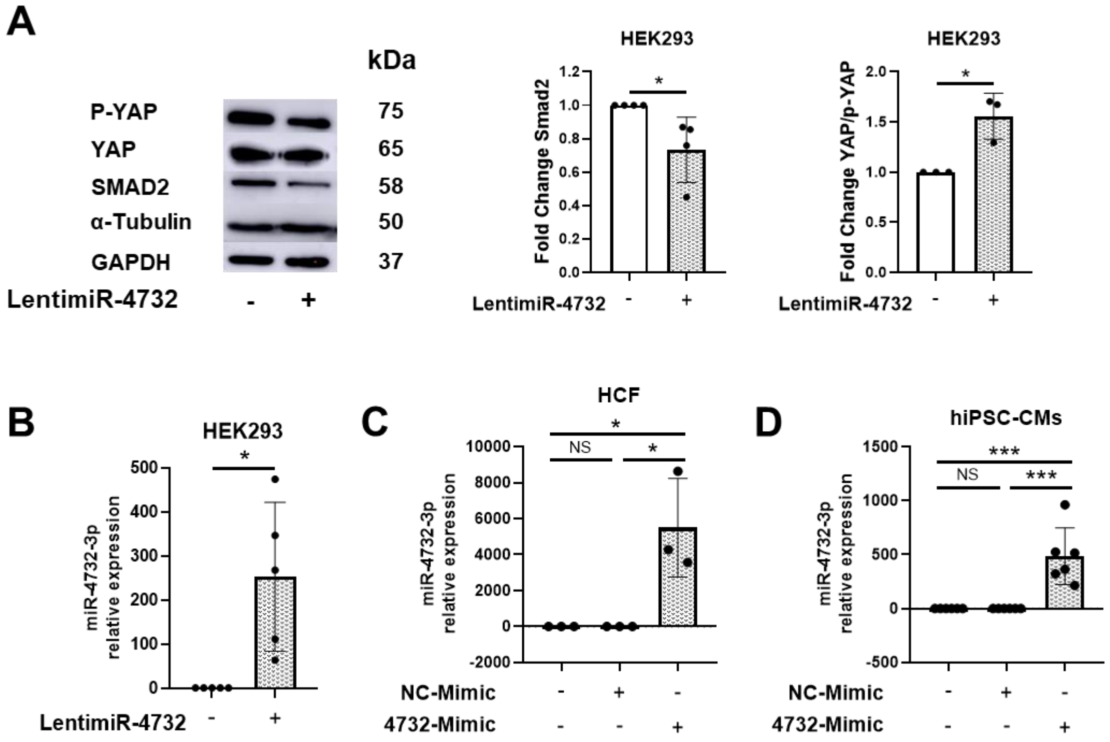

Figure S5. Identification and validation of miR-4732-3p target genes. (**A**) Representative Western blots of p-YAP, YAP, and Smad2 in HEK 293 non-infected or transduced with a lentivirus expressing miR-4732-3p. Relative levels were quantified by densitometry relative to the levels of HEK 293 without transduction. GAPDH was used as a loading control for p-YAP and YAP and α-tubulin was used as a loading control for Smad2. (**B**) Relative expression of miR-4732-3p in HEK 293 control or infected with the Lenti-miR-4732. (**C**) Relative expression of miR-4732-3p in HCF transfected using lipofectamine with either NC-Mimic or 4732-Mimic. (**D**) Relative expression of miR-4732-3p in hiPSC-CM transfected with either NC-Mimic or 4732-Mimic. Data are represented as mean ± standard deviation. NS = not significant *$p<0.05$, **$p<0.01$, ***$p<0.001$.
